# Tension pneumothorax caused by the ruptured hydatid cyst of the lung

**DOI:** 10.1002/ccr3.7542

**Published:** 2023-07-06

**Authors:** Reza Rezaei, Hossein Sadidi, Pegah Bahrami Taqanaki

**Affiliations:** ^1^ Endoscopic and Minimally Invasive Surgery Research Center, Ghaem Hospital Mashhad University of Medical Sciences Mashhad Iran; ^2^ Department of Thoracic Surgery, Ghaem Hospital Mashhad University of Medical Sciences Mashhad Iran; ^3^ Mashhad University of Medical Sciences Mashhad Iran

**Keywords:** cardiopulmonary arrest, case series, *Echinococcus granulosus*, hydatid cyst, tension pneumothorax

## Abstract

Hydatid cyst disease puts a significant burden on the health of humans every year. The lung is the second most common organ of implantation of *Echinococcus* larvae. Due to the importance of early diagnosis of tension pneumothorax, this paper provides four cases of hydatid disease that presented with tension pneumothorax.

## Graphical abstract

The chest CT scan of case 1 demonstrating the classic water‐lily sign.

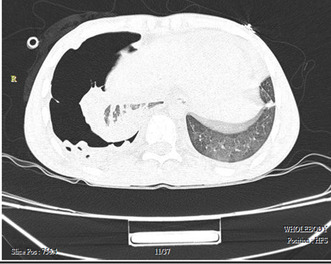

## 1 INTRODUCTION

Echinococcosis is a parasitic infection caused by larvae of a tapeworm genus called *Echinococcus*. The two main species of this genus causing significant problems in humans are *Echinococcus granulosus* and *Echinococcus multilocularis*, resulting in cystic and alveolar echinococcosis, respectively.^1^, ^2^ *E. granulosus* is relatively more common than *E. multilocularis* worldwide and also in Iran; however, *E. multilocularis* has been reported to be fatal, demanding a deliberate diagnostic approach.^3^ *E. granulosus* infection is endemic in China, Central Asia, South America, North and East Africa, Australia, and Eastern Europe.^4^ The life cycle of this parasite consists of definitive and final hosts (dogs) and intermediate hosts (herbivores such as sheep, cattle, camel, and goats).^5^ Humans are infected accidentally and are considered incidental hosts. The larvae of *E. granulosus* can reside in any organ with the liver followed by the lungs being the most common locations and other more uncommon sites include kidneys, brain tissue, spinal cord, spleen, pancreas, heart, adrenal glands, muscles, bones, ribs, and mediastinum.^6^ Patients remain asymptomatic until the cysts are either significantly enlarged or ruptured and then the symptoms are highly dependent on the organ involved.^5^ The special characteristics of the lung tissue as well as their negative pressure make them a suitable environment for the growth and placement of the cysts.^7^ Pulmonary manifestations of hydatid cyst disease are cough, chest pain, dyspnea, expectoration, fever, hemoptysis, allergic reactions, and rarely anaphylactic shock.^8^ The diagnostic approach to this disease includes taking a detailed history of the patient along with a complete physical examination, and after suspecting this disease, imaging modalities such as chest x‐ray and CT (computed tomography) scan of the chest and abdomen should be used. All patients with pulmonary hydatid cysts should be evaluated for liver cysts.^8^ Pulmonary hydatid cysts can be categorized based on their radiologic findings as small, giant, complicated, and uncomplicated.^7^, ^8^ Table 1 gives a summary of the classification.

**TABLE 1:** A summary of radiologic classification of the pulmonary hydatid cysts.

Classifications of hydatid cyst by radiology criteria	Small	Giant	Complicated	Uncomplicated
Features	Around 1 cm	>10 cm	Lesion with a well‐defined and smooth border	Ruptured or infected Radiologic features of ruptured hydatid cyst vary from air‐fluid to the water lily sign (occurring when cystic membranes float over the cystic fluid) and empty cyst sign (a fully drained cyst)

Pulmonary hydatid cysts can lead to life‐threatening complications, involving pulmonary artery embolism, pleural necrosis, pleural effusion, pneumothorax, tension pneumothorax, empyema, collapsed lung, and bronchopleural fistula.^9^ The treatment of this disease includes chemical treatment with mebendazole or albendazole and surgical treatment.^10^

Here we represent four cases of hydatid cysts of the lung manifesting as hydro‐pneumothorax and tension pneumothorax.

## 2 CASE PRESENTATION

### 2.1 Case 1

**FIGURE 1:**
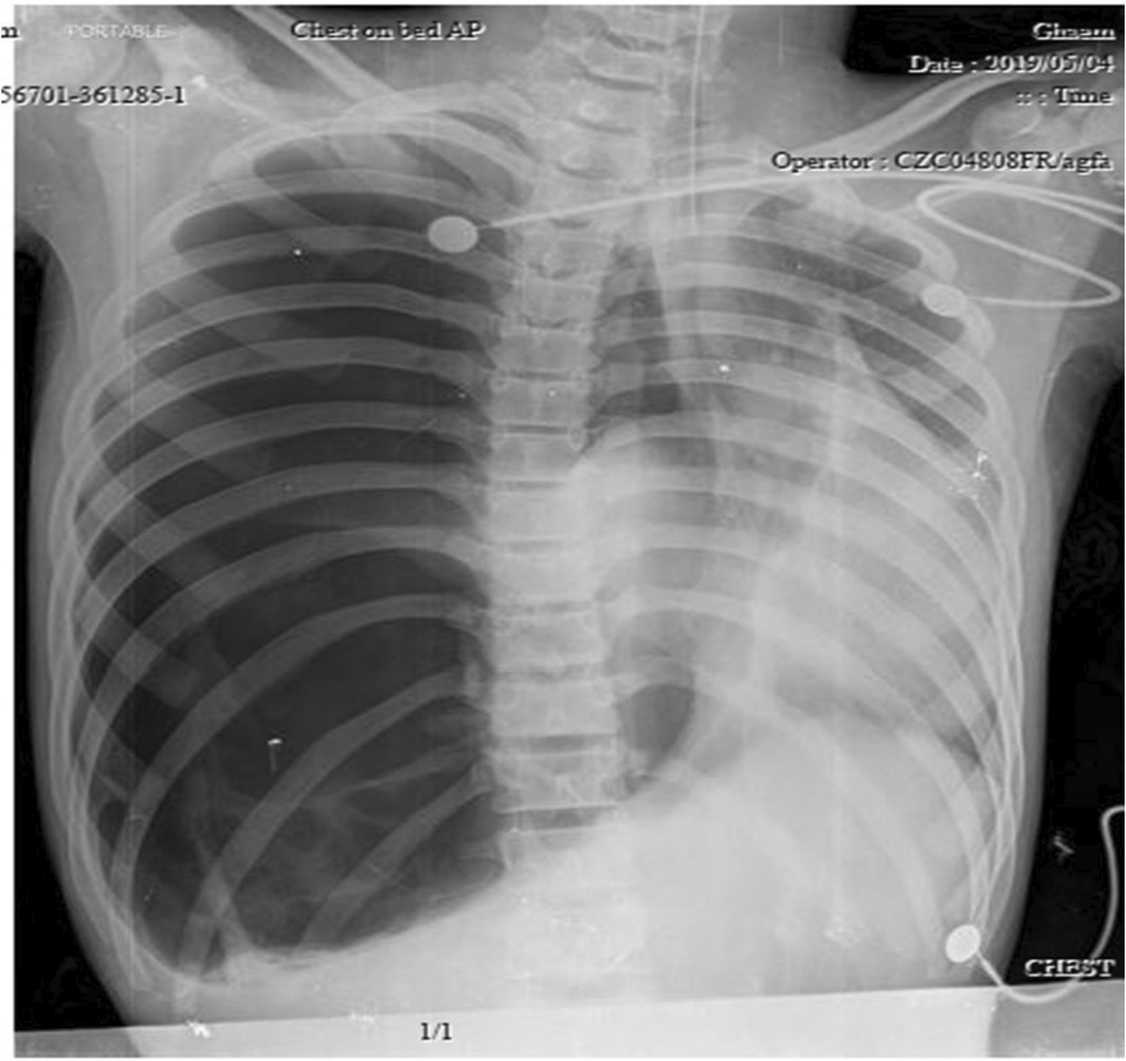
Collapse of the right lung indicating right‐sided tension pneumothorax in Case 1.

**FIGURE 2:**
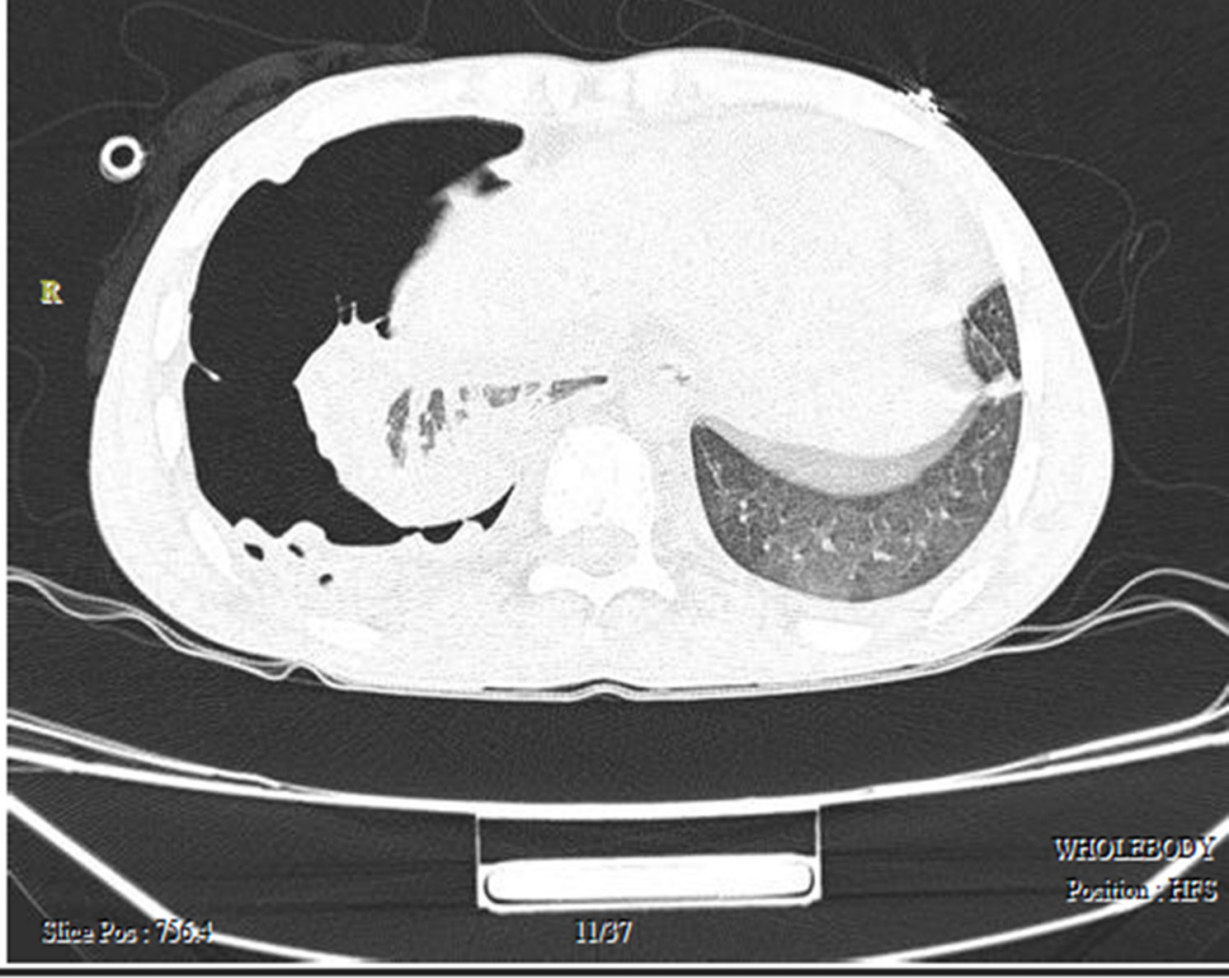
Chest CT scan of Case 1 demonstrating the classic water‐lily sign.

**FIGURE 3:**
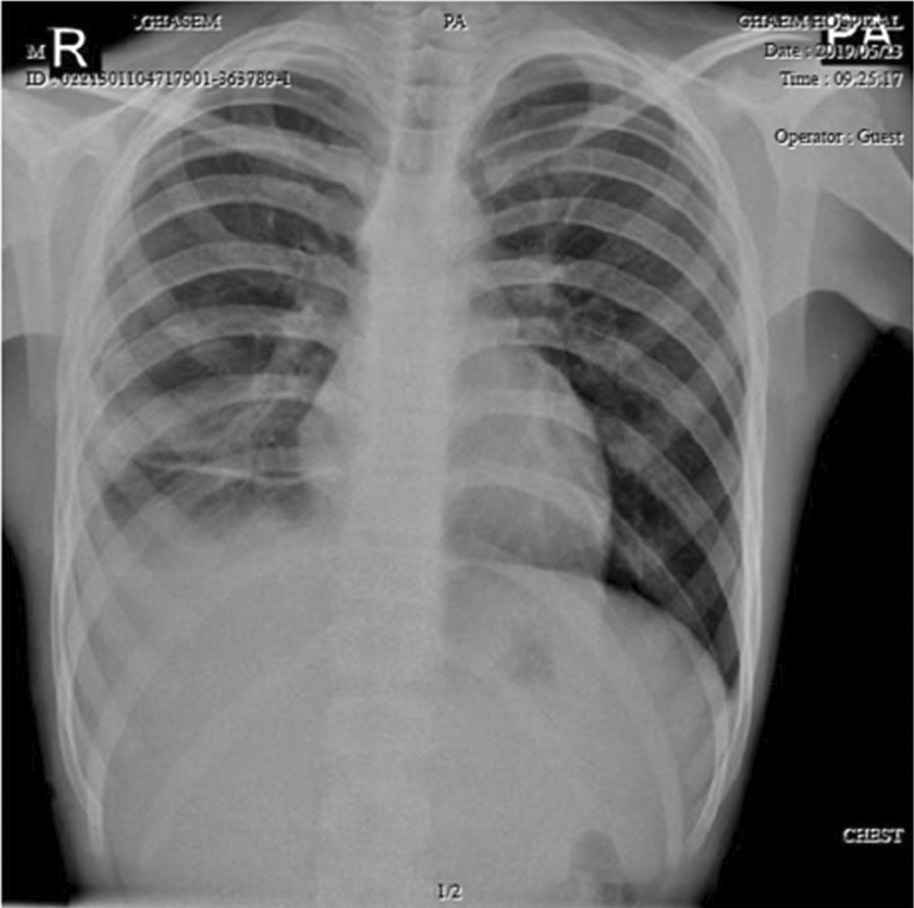
Postoperation chest x‐ray of Case 1 showing improvement and no recurrence.

A 16‐year‐old previously healthy male was admitted to the emergency department with sudden shortness of breath. Primary physical examination revealed hypotension (blood pressure = 70/50), tachycardia (pulse rate = 130), tachypnea (respiratory rate = 40), low oxygen saturation (SpO_2_ = 82%), and normal body temperature. While in the emergency room, he experienced cardiopulmonary arrest therefore resuscitation and intubation were carried out for the patient. After successful resuscitation, a portable chest x‐ray was done. Chest x‐ray findings indicated tension pneumothorax on the right side (Figure 1). Consequently, a chest tube was inserted to drain the excess air in the emergency department. After he gradually became more hemodynamically stable and his oxygen saturation increased (to SpO_2_ = 96%) a chest CT scan was performed, which showed the classic water lily sign (detached endocyst membranes of the hydatid cyst floating in cyst fluid) (Figure 2). The decision to do a right posterolateral thoracotomy was made. Intraoperative findings showed that the cyst membrane had fallen into the pleural space. After the removal of the membrane from the thoracic cavity, decortication was performed. The location of the hydatid cyst was detected to be in the right lower lobe and the site was then excised. After a full expansion of the right lung and removal of the chest tubes, he was discharged from the hospital with prescription of albendazole 600 mg daily. On follow‐up, he was symptom‐free, and no recurrence occurred (Figure 3).

### 2.2 Case 2

An 18‐year‐old female with no prior medical condition was referred to the emergency department with a 2‐month history of nonprogressive moderate exertional dyspnea accompanied by mild colicky pain in the epigastric area. Four days before admission, she had experienced severe pleuritic pain in the right hemithorax associated with dry cough, expectoration of salty fluid, and dyspnea which was also present at rest. Initial physical examination revealed normal blood pressure (blood pressure = 98/71), regular heart rate (pulse rate = 90), the axial temperature of 37°C, mild tachypnea (respiratory rate = 25), and absent breath sound in the right lung field.

**FIGURE 4:**
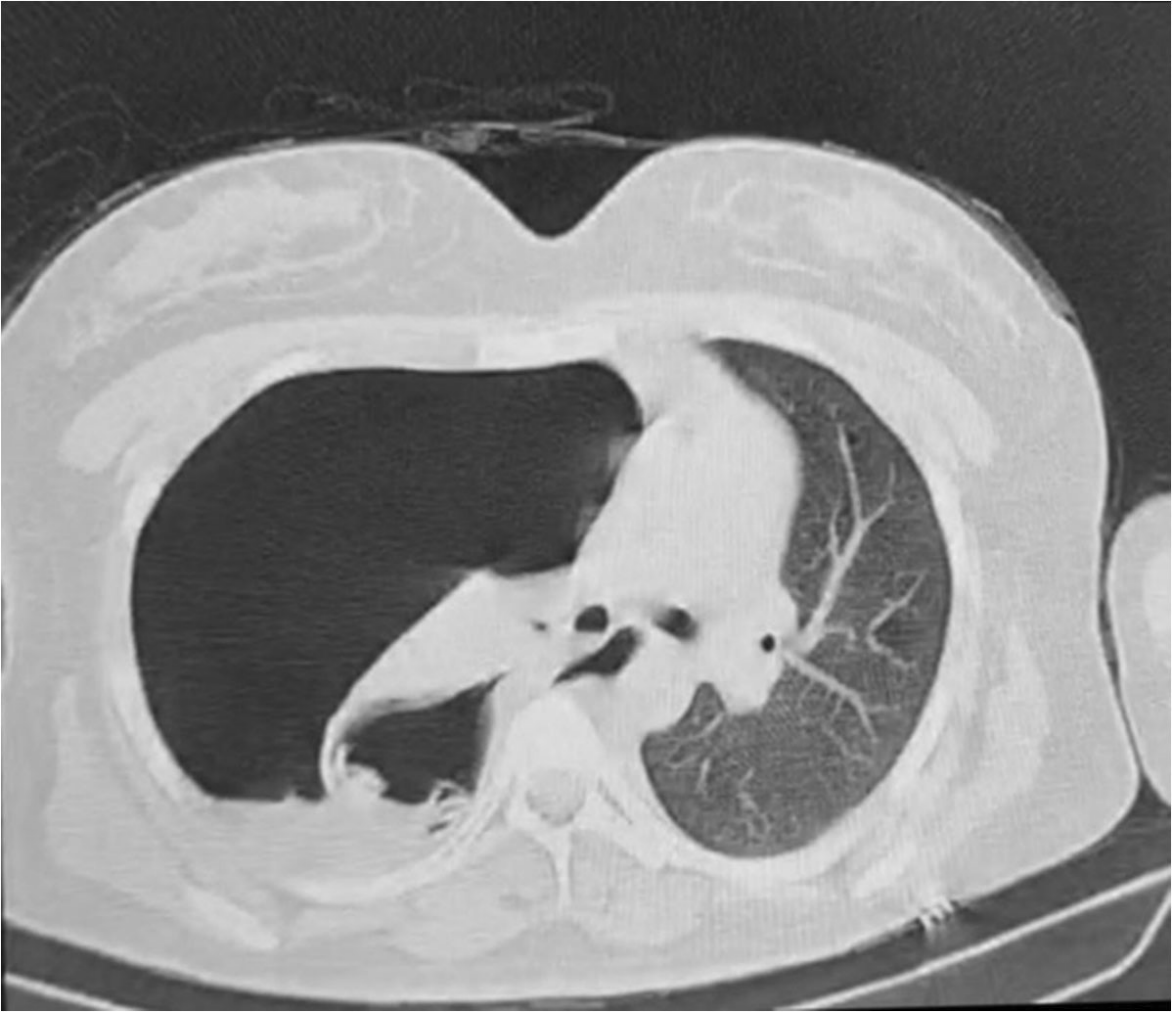
Chest CT scan of Case 2 indicating a mediastinal shift to the left side and floating membranes in the ruptured cyst on the right side.

Chest x‐ray findings included the total collapse of the right lung and shifting of the trachea and mediastinum to the left side; therefore, a CT chest scan was done which revealed severe hydro‐pneumothorax and two hydatid cysts localized in the superior lobe of the right lung and the left lobe of the liver which measured 43 × 54 mm and 68 × 88 mm, respectively (Figure 4). A few days after the placement of the chest tube and expansion of the lungs, with the diagnosis of a perforated hydatid cyst, the patient was a candidate for surgery. A right posterolateral thoracotomy was performed and the perforated hydatid cyst was resected. 7 days later; she was discharged in good general condition.

### 2.3 Case 3

**FIGURE 5:**
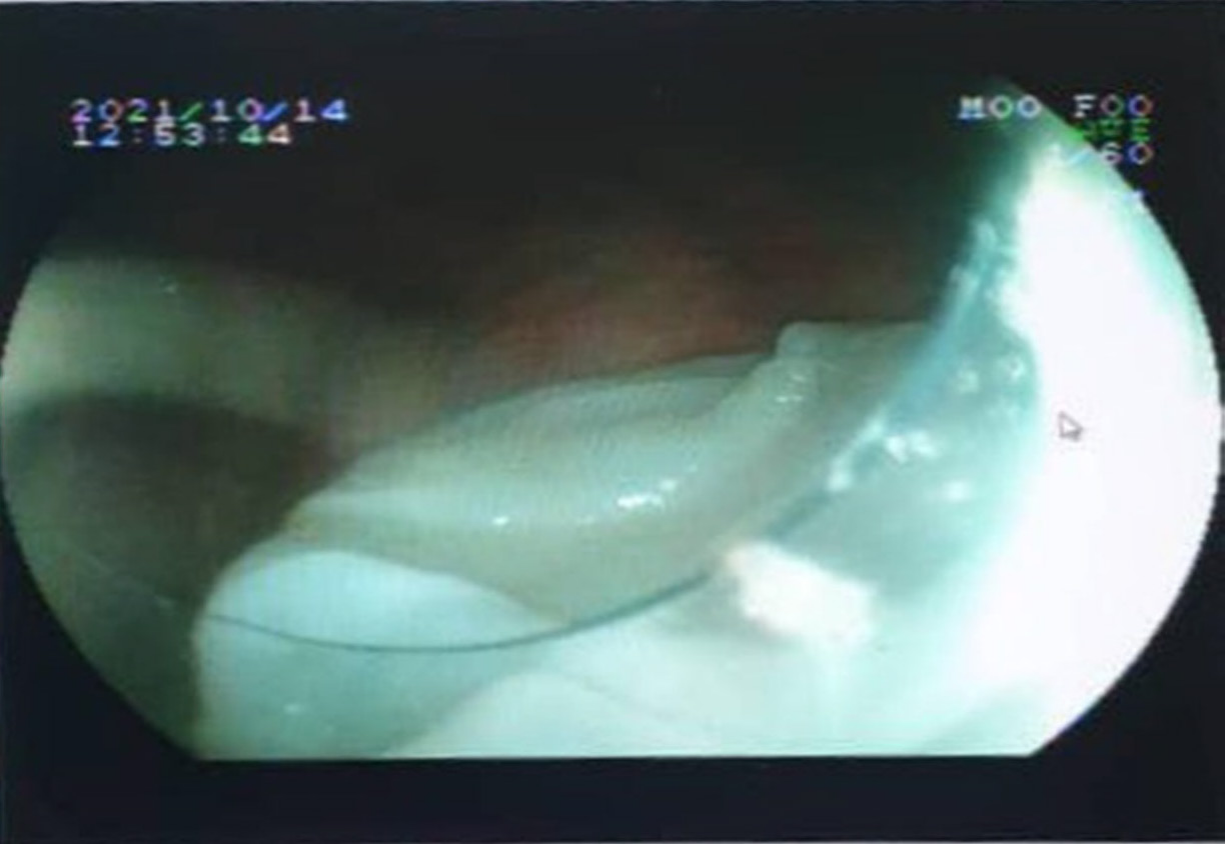
Image of the pleuroscopy performed in Case 3 revealing the perforated cystic membranes confirming the diagnosis of pulmonary hydatid cyst.

A 13‐year‐old boy with no underlying diseases was referred to the emergency department with the complaint of progressive intermittent dyspnea accompanied by coughing and discharge of bitter‐tasting liquid from the mouth. Further history‐taking revealed that 3 months prior to admission, the patient had experienced acute severe chest pain and dyspnea during playing football. Since then, his chest pain has improved, but exertional dyspnea remained. Upon arrival, the patient's vital signs were low blood pressure (blood pressure = 85/60), tachycardia (pulse rate = 130), tachypnea (respiratory rate = 30), and fever (38°C). On pulmonary auscultation, breath sounds were diminished on the left side of the chest. Chest radiography revealed the total collapse of the left lung and shifting of the trachea and mediastinum to the right side along a cyst‐like lesion. Chest CT was performed and confirmed the collapse of the left lung and mediastinal shift. Based on the patient's history and imaging findings and with the diagnosis of pulmonary hydatid cyst in mind, eventually, pleuroscopy was performed which revealed the perforated cystic membranes confirming the diagnosis of pulmonary hydatid cyst (Figure 5). The patient then underwent surgical removal of the hydatid cyst. He was put on treatment with Albendazole after surgery and was discharged a week later.

### 2.4 Case 4

**FIGURE 6:**
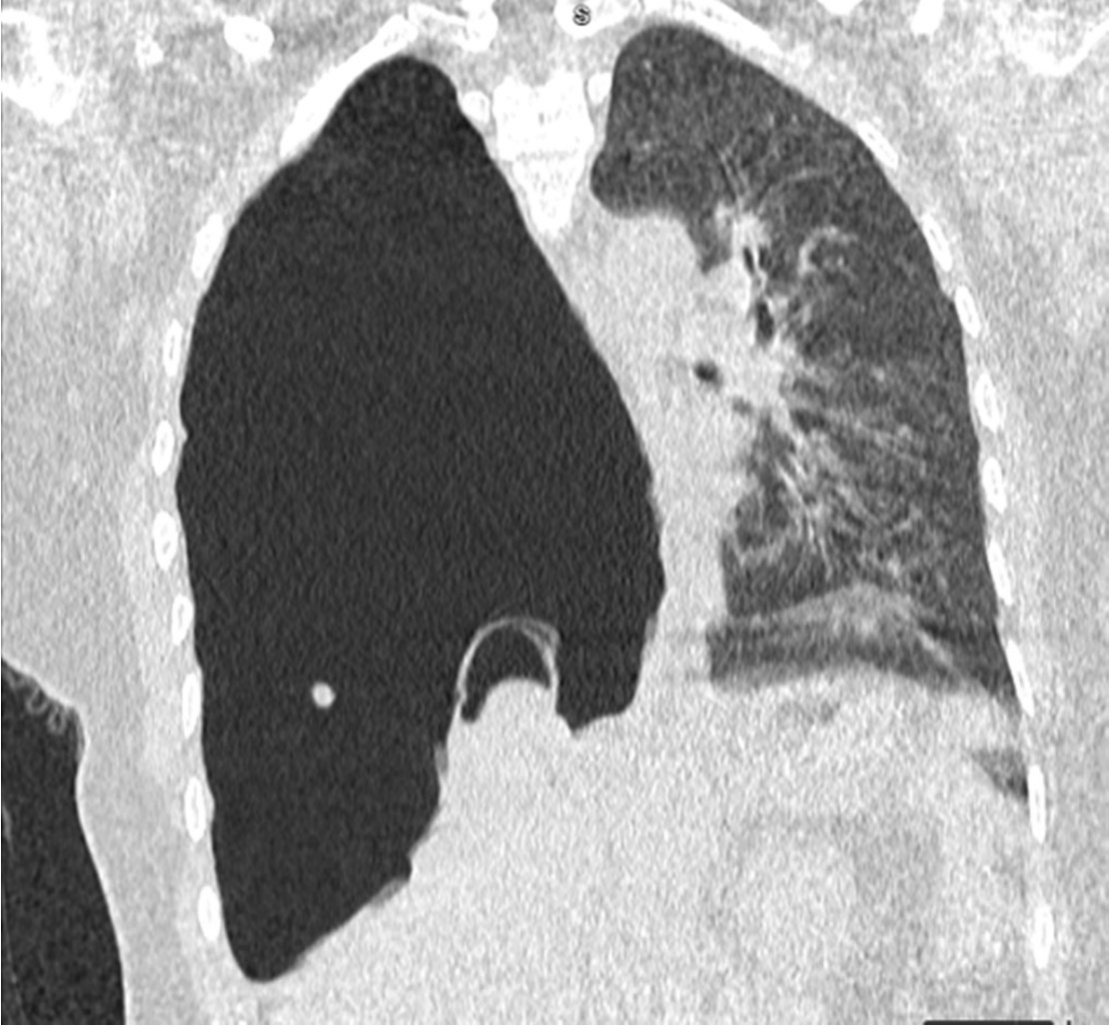
Chest x‐ray of Case 4 indicating a cystic lesion in the lower region of the right lung.

**FIGURE 7:**
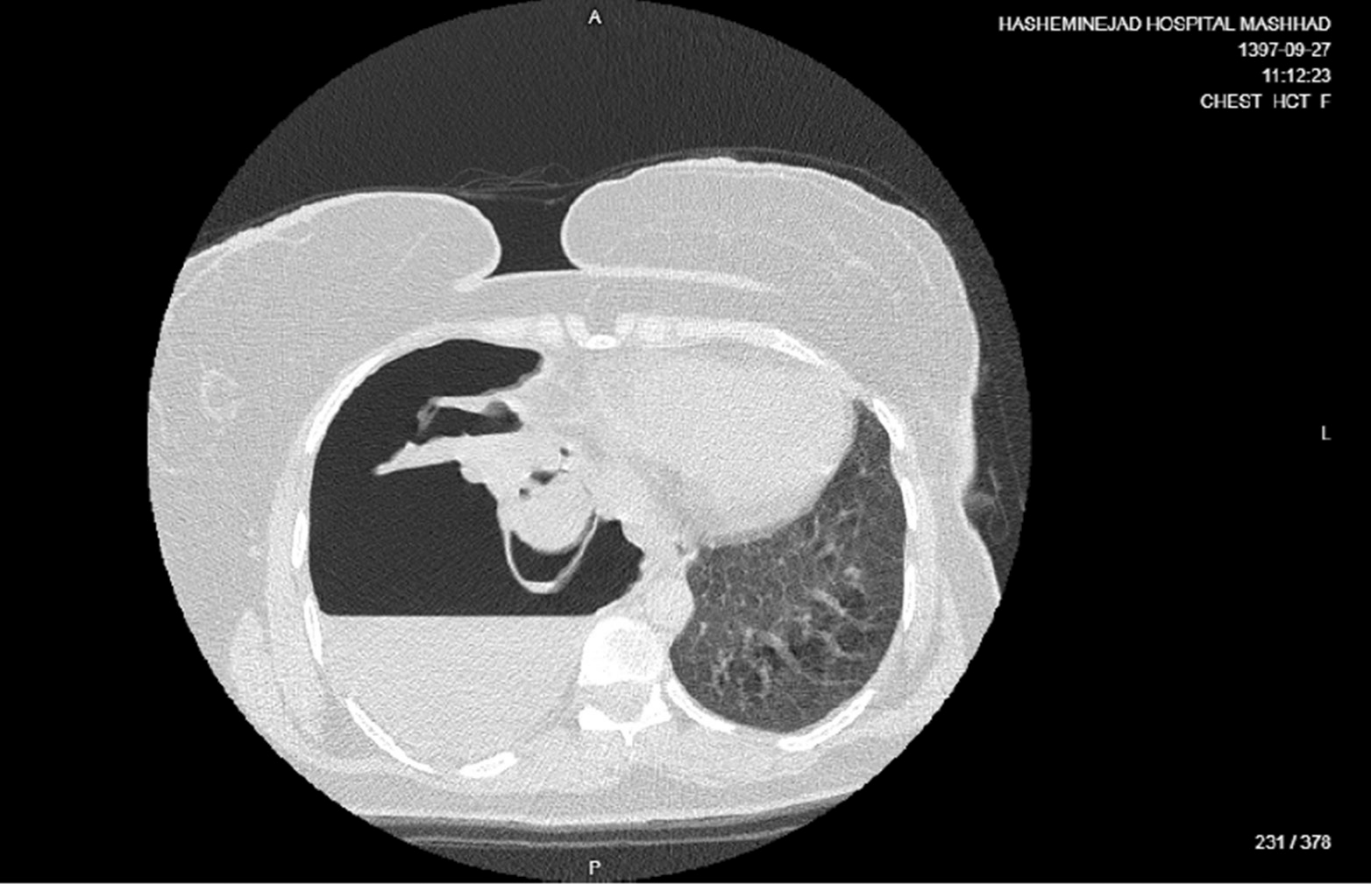
Chest CT scan of Case 4 revealed a collapsed right lung, mediastinal shift to the left side, and a cystic lesion resembling a hydatid cyst with a ruptured membrane.

**FIGURE 8:**
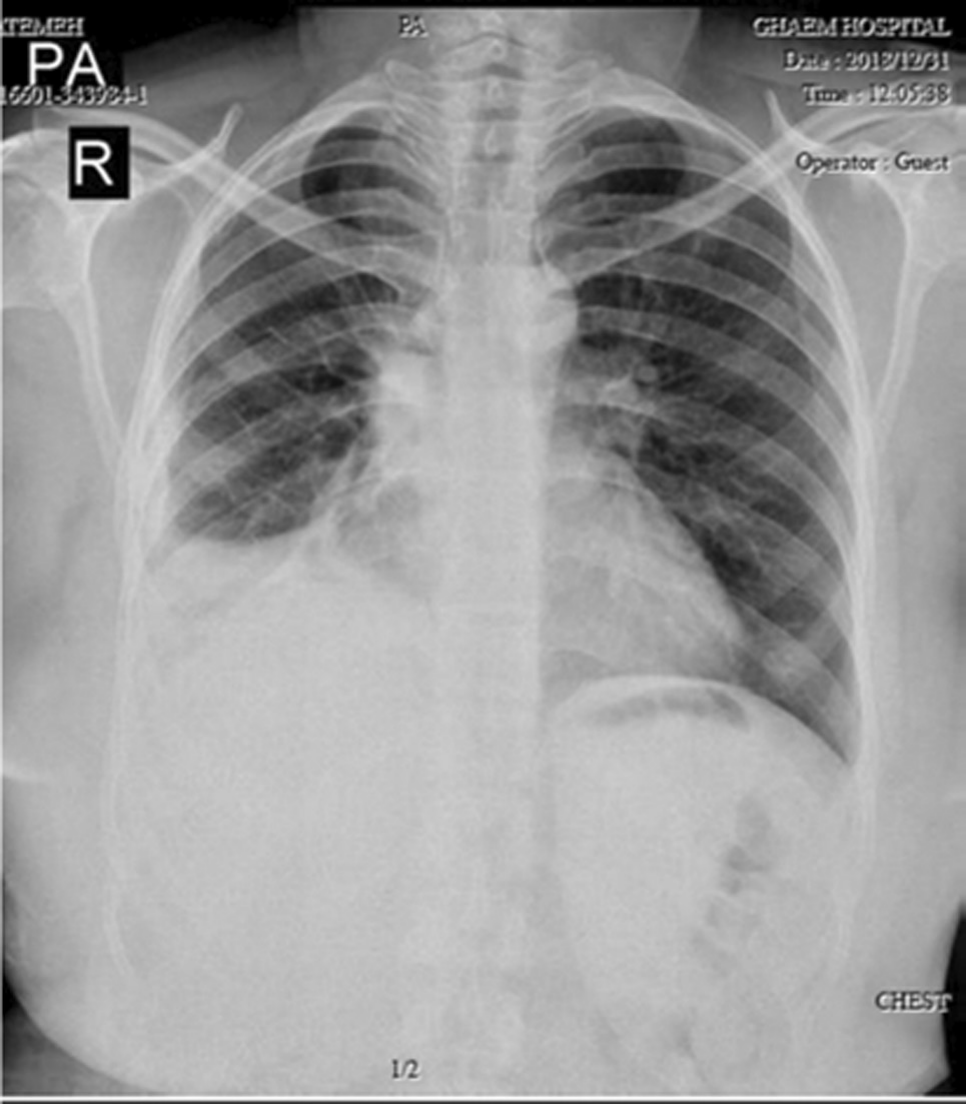
The follow‐up chest x‐ray of Case 4 showing no abnormalities.

A 25‐year‐old female with no past medical history presented to a trauma center with acute dyspnea. On examination, she had hypotension (blood pressure = 85/70), tachycardia (Pulse rate = 119), tachypnea (respiratory rate = 25), and fever (38.5°C). On auscultation, no breath sounds were heard on the right side of the chest. With pneumothorax in mind, a portable chest x‐ray was performed revealing collapse of the right lung and mediastinal shift to the left side confirming the diagnosis. The chest x‐ray also demonstrated a cystic lesion in the lower region of the right lung (Figure 6). Chest CT showed a collapsed right lung, a mediastinal shift to the left side, and a cystic lesion resembling a hydatid cyst with a ruptured ectocystic layer (Figure 7). A chest tube was inserted and subsequently, the patient's breathing improved a little. She later underwent surgery and intraoperative findings indicated massive destruction of the lung tissue so a complete lower lobectomy of the right lung was performed. Postoperatively her breathing improved. She was discharged after extubation and medical therapy with albendazole was continued after discharge. On follow‐up, she was doing well and her chest x‐ray showed no abnormalities (Figure 8).

## 3 DISCUSSION

**FIGURE 9:**
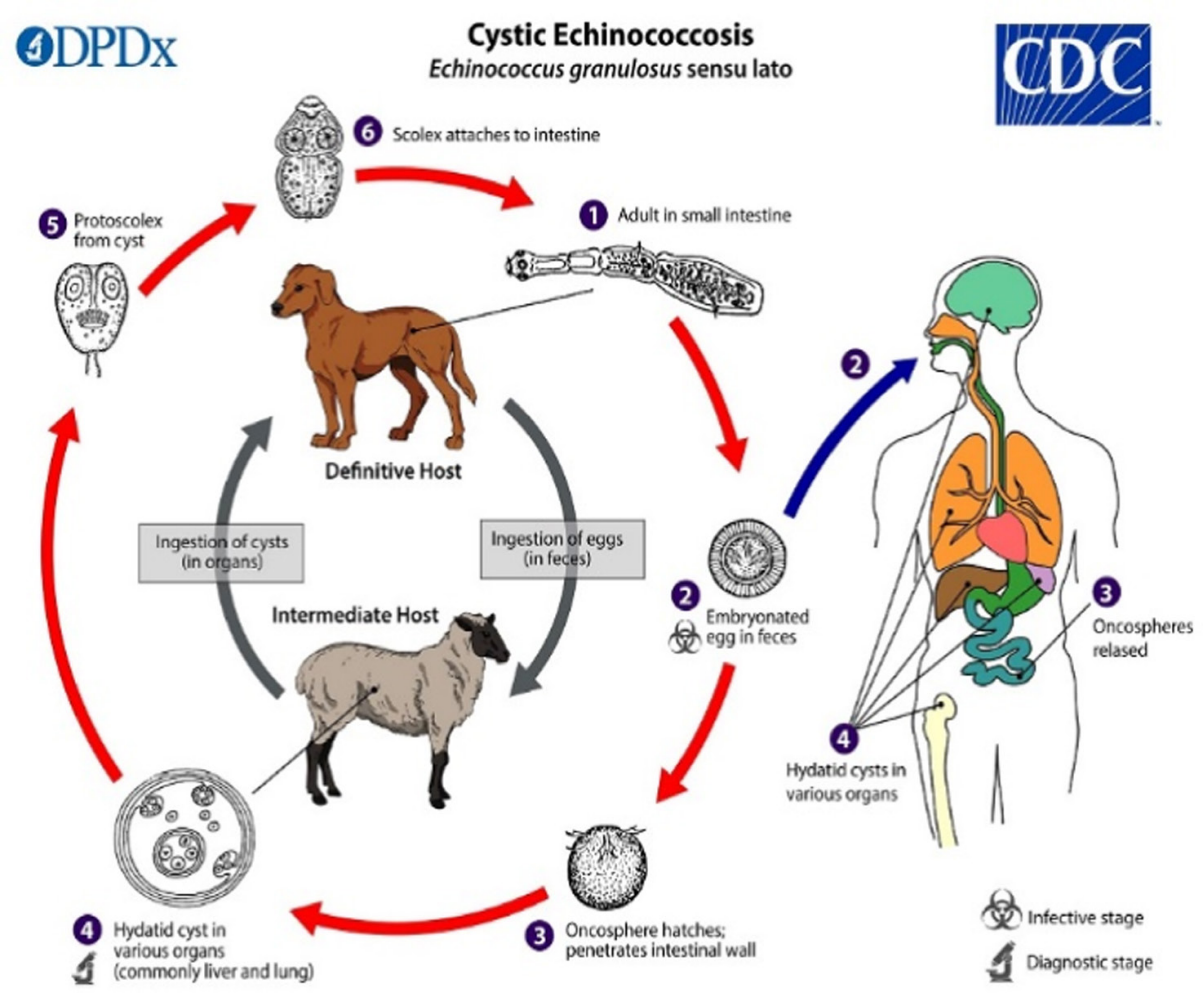
The life cycle of *Echinococcus granulosus*. (Image obtained from the Centers for Disease Control and Prevention Image Library, Global Health, Division of Parasitic Diseases and Malaria.)

Hydatid disease, as a common disease in areas with livestock, has always been a point of concern for physicians due to the complications it may have in the long term. After humans are infected with *Echinococcus*, this organism spreads to other organs by invading the intestinal mucosa.^8^ Figure 9 demonstrates the life cycle of *E. granulosus*. Pulmonary hydatid cysts as well as cysts in any other organs remain asymptomatic until enlarged or ruptured. Rupture in lung cysts may occur due to any condition leading to a rise in pulmonary or intra‐abdominal pressure.^11^ Tension pneumothorax is a rare complication of the pulmonary hydatid cyst and if acute it can manifest as severe dyspnea, tachycardia, tachypnea, jugular vein distention, and hypotension with unilateral diminished lung sounds, and trachea‐mediastinal shift to the opposite side.^11^

In our cases, symptoms such as intermittent dyspnea which had gradually worsened over time, accompanied by cough and expectoration of salty fluid, along with the hydatid disease being endemic in our region led us to suspect hydatidosis as the cause. In all four of our patients, pulmonary sounds were unilaterally absent. Examination and imaging findings indicated massive hydro‐pneumothorax and tension pneumothorax. Imaging modalities such as CT scan and chest x‐ray were of great help in confirming the diagnosis of tension pneumothorax and ruptured hydatid cyst. By observing the ruptured membranes and water lily sign, the diagnostic suspicion of a pulmonary hydatid cyst was confirmed. And finally, intraoperative findings demonstrated hydatid cyst as the definite cause and the pathology examination of the tissue also confirmed this diagnosis.

There have been a number of other cases of tension pneumothorax caused by a ruptured hydatid cyst in Nepal, Iraq, Israel, and Turkey^11^, ^12^, ^13^, ^14^ and a summary of these cases is presented in Table 2.

**TABLE 2:** Review of reported cases of tension pneumothorax caused by hydatid cyst disease.

Author	Number of patients	Age/sex	Signs and symptoms	Imaging	Management	Outcome and follow‐up
Acharya et al. 2020^11^	1	31 Male	Dyspnea, pleuritic chest pain on left side, hemoptysis, fever, tachypnea, low SpO_2_ (90%), tracheal shift to the left, hyper‐resonance on percussion	Cavity with thick walls in left lower lobe with air fluid level and multiple membrane‐like structures within the cavity suggestive of ruptured hydatid cyst	Needle thoracostomy, surgical resection of the cyst, pleurodesis	Complete recovery
Bakir et al. 1969^12^	5	12–40 Male (1), females (4)	Dyspnea and cough (4 patients), chest pain (5 patients), expectoration of salty fluid (2 patients), fever and tachypnea (4 patients), tracheal shift and tachycardia (5 patients)	Collapse of lung (4 patients), air‐fluid level (2 patients), hydro‐pneumothorax (1 patient), pneumothorax (1patient), water‐lily sign (1 patient)	Chest aspiration or water‐seal was done in all patients, cyst removal (3 patients, which was accompanied by closing a broncho‐pleural fistula in one of them), lobectomy (1patient)	Four patients recovered fully with no recurrence but the last patient who was a pregnant woman suffered from the infection due to the infected lung cavity. The long‐term follow up of the patent was not mentioned in the article.
Sharon et al. 2012^13^	1	38 Male	Dyspnea, hemoptysis pleuritic chest pain at first and was diagnosed with lung hydatid cyst but refused surgery and presented with these symptoms a week later: recurrent syncope, hypotension, JVP distention, and diminished breath sounds on the right side	First CT scan showed findings indicative of a lung hydatid cyst (findings not specified), massive pleural effusion compressing the superior vena cava and heart	Drainage of empyema, due to the patient's refusal of surgery he was put on IV antibiotics (piperacillin/tazobactam and clindamycin)and oral albendazole	Imaging 2 years later showed empty cyst with broncho‐fibrosis
Yekeler et al. 2009^14^	1	26 Male	Progressive dyspnea and chest pain after blunt trauma, cough, expectoration of salty fluid, hypotension, tachypnea, tachycardia	Ruptured cyst in the left hemithorax, cardiac shift to the right, and free fluid	Chest tube insertion, thoracotomy with removal of cyst and oral therapy with albendazole	Complete recovery
Sahin et al. 2012^15^	1	4 Male	Dyspnea, coughing, fever	Left‐sided tension pneumothorax and a cystic lesion in the left hemi‐thorax	Chest tube insertion, resection of the cyst with capitonnage, and decortication	Complete recovery

The initial treatment for tension pneumothorax accompanied by unstable hemodynamics is needle thoracostomy followed by chest tube insertion. When tension pneumothorax is caused by a ruptured pulmonary hydatid cyst, the main treatment is surgery with the aim of resecting the hydatid cysts without contamination of the pleural and mediastinal cavities.^11^ Surgical techniques consist of enucleation (total removal of cyst), pericystectomy (removal of the first layer of the cyst), cystotomy with or without capitonnage of the pericystic space, and radical pulmonary resection.^11^ Therapy with an antiparasitic agent decreases the risk of recurrence in the patients. It is recommended to prescribe a three‐phased treatment plan with antiparasitic agents with each phase including 6 weeks of treatment and 2 weeks of no medication.^16^

In conclusion, tension pneumothorax caused by a ruptured hydatid cyst is not common. Hydatid cysts usually affect the liver, but they can also impact the lungs, which may result in serious complications such as tension pneumothorax and hydropneumothorax. As hydatid cyst disease causes a significant burden on human health, it must be considered as a differential diagnosis, especially in endemic areas, in patients presenting with dyspnea and tension pneumothorax.

Accompanying symptoms including expectorations of salty fluid and a history of blunt chest trauma prior to symptoms should be taken seriously by clinicians. Imaging assessments including chest x‐ray and CT scans are helpful. Treatment of a ruptured lung hydatid cyst causing tension pneumothorax is mainly surgery. Therefore, it is crucial to gather a comprehensive medical history and determine the underlying cause of pneumothorax, as the management of hydatid cyst‐induced pneumothorax differs from other causes. Additionally, patients require antiparasitic treatment to prevent a recurrence.

## AUTHOR CONTRIBUTIONS

**Reza Rezaei:** Conceptualization; data curation; investigation; methodology; project administration; supervision. **Hossein Sadidi:** Data curation; resources; writing – original draft; writing – review and editing. **Pegah Bahrami Taqanaki:** Data curation; visualization; writing – original draft; writing – review and editing.

## FUNDING INFORMATION

This research did not receive any specific grant from funding agencies in the public, commercial, or not‐for‐profit sectors.

## CONFLICT OF INTEREST STATEMENT

The authors declare that they have no competing interests.

## ETHICS STATEMENT

This study has been approved by the Mashhad University of Medical Sciences ethical committee and is in line with the Declaration of Helsinki.

## CONSENT

Written informed consent was obtained from the patient to publish this report in accordance with the journal's patient consent policy. Permission to reproduce material from other sources: Not applicable.

## Data Availability

All data underlying the results are available as part of the article and no additional source data are required.

## References

[1] Dong Z , Yusup M , Lu Y , Tang B . Hydatid cyst of the heart as a rare cause of arrhythmia: a case report and review of published reports. HeartRhythm Case Rep. 2022;8:458‐462. 35774212 10.1016/j.hrcr.2022.04.004 PMC9237349

[2] Casulli A , Barth TFE , Tamarozzi F . Echinococcus multilocularis . Trends Parasitol. 2019;35(9):738‐739. 31182385 10.1016/j.pt.2019.05.005

[3] Mahmoudi S , Mamishi S , Banar M , Pourakbari B , Keshavarz H . Epidemiology of echinococcosis in Iran: a systematic review and meta‐analysis. BMC Infect Dis. 2019;19(1):1‐19. 31684882 10.1186/s12879-019-4458-5 PMC6830007

[4] Tamarozzi F , Legnardi M , Fittipaldo A , Drigo M , Cassini R . Epidemiological distribution of *Echinococcus granulosus* s. l. Infection in human and domestic animal hosts in European Mediterranean and Balkan countries: a systematic review. PLoS Negl Trop Dis. 2020;14(8):e0008519. 32776936 10.1371/journal.pntd.0008519 PMC7440662

[5] Vaisi‐Raygani A , Mohammadi M , Jalali R , Salari N , Hosseinian‐Far M . Prevalence of cystic echinococcosis in slaughtered livestock in Iran: a systematic review and meta‐analysis. BMC Infect Dis. 2021;21(1):1‐10. 33962578 10.1186/s12879-021-06127-2 PMC8103583

[6] Aghajanzadeh M , Jalal A , Saffar M , Youseffi S , Mousavi M , Habibzadeh K . Reports of symptoms, diagnosis and management of eight cases of primary and isolated splenic hydatid cyst. JSM Clin Cytol Pathol. 2019;4(1/5).

[7] Durhan G , Tan AA , Düzgün SA , Akkaya S , Arıyürek OM . Radiological manifestations of thoracic hydatid cysts: pulmonary and extrapulmonary findings. Insights Imaging. 2020;11(1):116. 33175295 10.1186/s13244-020-00916-0 PMC7658283

[8] Rawat S , Kumar R , Raja J , Singh RS , Thingnam SKS . Pulmonary hydatid cyst: review of literature. J Family Med Prim Care. 2019;8(9):2774‐2778. 31681642 10.4103/jfmpc.jfmpc_624_19 PMC6820383

[9] Saeedan MB , Aljohani IM , Alghofaily KA , Loutfi S , Ghosh S . Thoracic hydatid disease: a radiologic review of unusual cases. World J Clin Cases. 2020;8(7):1203‐1212. 32337194 10.12998/wjcc.v8.i7.1203 PMC7176618

[10] Pal M , Alemu HH , Marami LM , Garedo DR , Bodena EB . Cystic Echincoccoosis: A Comprehensive Review on Life Cycle, Epidemiology, Pathogenesis, Clinical Spectrum, Diagnosis. Public Health and Economic Implications, Treatment, and Control; 2022.

[11] Acharya AB , Bhatta N , Mishra DR , Verma A , Shahi R . Rare cause of tension pneumothorax: hydatid disease of lung: a case report. JNMA J Nepal Med Assoc. 2020;58(224):265. 32417867 10.31729/jnma.4693 PMC7580465

[12] Bakir F , Al‐Omeri MM . Echinococcal tension pneumothorax. Thorax. 1969;24(5):547‐556. 5348321 10.1136/thx.24.5.547 PMC472048

[13] Sharon H , Elhanan E , Aviram G , Hassin D . Tension pyopneumothorax due to a ruptured pulmonary echinococcal cyst. Respiration. 2012;84(4):327‐328. 22869518 10.1159/000339510

[14] Yekeler E , Celik O , Becerik C . A giant ruptured hydatid cyst causing tension pneumothorax and hemothorax in a patient with blunt thoracic trauma: a rare case encountered in the emergency clinic. J Emerg Med. 2012;43(1):111‐113. 19592190 10.1016/j.jemermed.2009.04.066

[15] Sahin E , Karadayi S , Nadir A , Kaptanoglu M . Spontaneous enucleated hydatid cyst with tension pneumothorax: a case report. Turk J Thorac Cardiovasc Surg. 2012;20(1):166‐168.

[16] Fattahi Masoom SH , Lari SM , Fattahi AS , Ahmadnia N , Rajabi M , NaderiKalat M . Albendazole therapy in human lung and liver hydatid cysts: a 13‐year experience. Clin Respir J. 2018;12(3):1076‐1083. 28319358 10.1111/crj.12630

